# Primary Care Patient Records in the United Kingdom: Past, Present, and Future Research Priorities

**DOI:** 10.2196/11293

**Published:** 2018-12-19

**Authors:** Brian McMillan, Robert Eastham, Benjamin Brown, Richard Fitton, David Dickinson

**Affiliations:** 1 Centre for Primary Care and Health Services Research, Division of Population Health, Health Services Research and Primary Care School of Health Sciences, Faculty of Biology, Medicine and Health University of Manchester Manchester United Kingdom; 2 Whitehall Surgery Wortley Beck Health Centre Lower Wortley Leeds United Kingdom; 3 Centre for Health Informatics, Division of Informatics, Imaging & Data Sciences School of Health Sciences, Faculty of Biology, Medicine and Health University of Manchester Manchester United Kingdom; 4 West Pennine Local Medical Committee Barley Clough Medical Centre Nugget Street Oldham United Kingdom; 5 Unlike Minds Gorton Monastery Manchester United Kingdom

**Keywords:** primary care, access to records, medical records, computerized records

## Abstract

This paper briefly outlines the history of the medical record and the factors contributing to the adoption of computerized records in primary care in the United Kingdom. It discusses how both paper-based and electronic health records have traditionally been used in the past and goes on to examine how enabling patients to access their own primary care record online is changing the form and function of the patient record. In addition, it looks at the evidence for the benefits of Web-based access and discusses some of the challenges faced in this transition. Finally, some suggestions are made regarding the future of the patient record and research questions that need to be addressed to help deepen our understanding of how they can be used more beneficially by both patients and clinicians.

## A Brief History of the Medical Record

The history of medical records can be dated back as far as the Edwin Smith papyrus of 1600 BC, which describes 48 surgical case histories and was most likely written as an Egyptian surgical manual [1]. Later examples include the case histories of Hippocrates from around 400 BC [2] and medieval Islamic texts from around AD 925, which were largely adapted from Graeco-Roman case histories [3]. Throughout the centuries, medical records were mainly used for teaching purposes [4], and the popularity of cadaveric dissection in the 17th century focused on the use of case histories for the teaching of anatomy [5]. By the 1700s, the keeping of case history books by physicians was becoming more commonplace [6], and medical centers were keeping increasingly detailed patient records toward the end of that century and into the 1800s [7,8]. In the late 1800s, attempts were made to control the content and quality of hospital records for insurance and medicolegal purposes [7], but it was common at this time for physicians to keep their private notes separately to aid patient care [4].

In United Kingdom, Lloyd George’s National Insurance Act of 1911 made it compulsory for employed men aged 16-70 years to take out health insurance, and for general practitioners (GPs) providing their care to keep a written record of these patients [9]. While the content and layout of the record were not stipulated, their size was determined by the tin storage boxes provided by the government at that time [10]. These metal boxes were later replaced by envelopes, but the size of the primary care record persisted after the introduction of the National Health Service (NHS) in 1948 [10]. Early criticisms of the format of general practice records focused on the inconvenience caused by the small size of the envelopes, and the absence of a separate problems list [10]. To overcome these problems, there were calls for primary care surgeries to change to records in an A4 format in the 1960s and 1970s, but these failed to materialize [10]. Such concerns were soon to be made redundant by the introduction of computerized records systems [9].

## Transition to Electronic Records

The history of computerized records in general practice can be traced back to Exeter in 1970 when John Preece became the first GP to use a computer in the consulting room [11]. The first government-sponsored electronic records system involved a small pilot by the Department of Health in Exeter in 1972 [9]. Ten years later, the government-sponsored “Micros for GPs” involving 150 UK practices, laying the foundations for further innovations [9]. In 1987, 2 private companies began offering computer systems to general practices free of charge with a plan to offer anonymized data to pharmaceutical companies to recoup their initial investment [11]. These schemes were hugely popular with GPs and this, coupled with remuneration changes in 1990, resulted in an exponential growth in the number of GP practices using computerized systems [9]. While <5% of GP practices used electronic records in the early 1980s, this increased to 80% in 1992 as government incentives continued [9] and by 1996, 96% of general practices used computerized record systems [11].

## Evolving Functions of the Electronic Record

While the functions of the paper-based patient record expanded slowly over the centuries, the computerization of medical records in primary care has opened up a wealth of additional functionality. The functions of the electronic patient record can be roughly categorized into clinical, administrative, and statistical, although there is some degree of overlap. The electronic record continues to be used primarily as a clinician’s aide memoir, enabling primary care staff to see what was discussed at previous appointments or refer to a list of patients’ current and previous medical problems. Clinical tasks, such as prescribing, have become easier, safer, and more cost-efficient as electronic record systems can flag allergies, contraindications, potential drug interactions, and suggest lower cost-generic alternatives. Some electronic record systems link to knowledge databases, such as the National Institute of Health and Care Excellence Clinical Knowledge Summaries, or provide handy links to patient information leaflets such as those hosted on “patient.info.” Computerized records make it easier to ensure patients are followed up in a timely manner through the use of a “recall” function. Clinical audits can be carried out at the push of a button, enabling clinicians to ascertain how patient care can be improved, or identify patients who are slipping through the net.

In addition, administrative tasks are now vastly less labor-intensive. Keeping an up-to-date list of patients containing accurate demographic and clinical information no longer requires meters of filing cabinet; letters to patients and other specialties can be prepopulated with important information from a patient’s record; and patient record transfers between GP surgeries is now increasingly an electronic process. Moreover, electronic record systems are used in the financial management of practices, for purposes such as securing reimbursement, budget planning, and reducing costs. Furthermore, the electronic patient record system can be used to enable secure communication between members of staff, reducing the risk of tasks being left undone and with the added benefit of an audit trail.

Computerized primary care records also provide a wealth of statistical information. The UK government has long seen the potential value of collecting such information [10], and there have been ill-fated attempts to monetize this information in the past by private companies [11]. The early GP computer enthusiasts designed computer systems to collect epidemiological data, and this tradition has continued to this day. Research using the Clinical Practice Research Datalink, which holds data on over 11.3 million patients from 674 UK practices [12], has resulted in a multitude of improvements in patient care and over 1800 scientific publications [13]. There is a growing interest in using machine learning approaches to define disease phenotypes in electronic primary care health records [14] while others are using statistical techniques used in astrophysics to develop predictive models of disease from the Clinical Practice Research Datalink [15].

In addition, the patient record can now be used by clinicians to send referrals directly to secondary care. Standardizing information flow between referrer and service provider is becoming an increasingly important function of clinical systems. A 2016 audit of suspected cancer referrals in Leeds found that only 48% were completed with the minimum required clinical information; this can lead to a delay in investigation and diagnosis. By leveraging existing functionality within SystmOne, the “DART” project to streamline the referrals process led to 100% of forms completed correctly within 3 months of introduction [16].

Projects such as “DART” illustrate how clinical systems have the potential to both improve patient safety and free-up much needed clinical resources. However, some initiatives to improve patient outcomes by harnessing the functionality within clinical systems may conversely have a detrimental impact on GP workload. The 2016 King’s Fund report aimed at “Understanding pressures in general practice” [17] cited the potential for new preventive services to impact the GP workload negatively. Preventive services (such as monitoring of chronic disease) have largely been made possible by recent advances in clinical systems. However, by linking chronic disease management functions to Quality and Outcomes Framework targets, there is an inevitable pressure for a huge amount of information to be manually read-coded within the record. Failure to do so can have a direct impact on practice income. Mindful of these tensions, it would seem imperative that future initiatives to use clinical systems to improve patient outcomes must take great care not to impact a clinician’s workload adversely.

## Enabling Patients’ Access to Their Own Records

Throughout history, the medical record has traditionally primarily served clinicians and served patients only indirectly. The idea of enabling patients to have full access to their medical record, however, is not entirely new. For example, in 1973, Shenkin and Warner noted,

Dissatisfaction with the functioning of the medical care system has become widespread. Four serious problems are maintaining high quality of care, establishing mutually satisfactory physician-patient relations, ensuring continuity and avoiding excessive bureaucracy. We believe these problems could be alleviated, in part, if patients were given copies of all their medical records.

18, p 688

Early proponents of granting patients open access to their primary care record included GPs from Balsall Health Centre in Birmingham who started enabling patients to access their full primary care record in 1977 [19], and GPs from Wells Park Road Practice in London who enabled full access from 1983 [20]. Reviews of the impact of promoting such access have shown beneficial effects and minimal risks [21].

The introduction of the Data Protection Act in 1998 gave patients the legal right to access their health records [22], setting the scene for changes to come. While the patient records aspect of the NHS Connecting for Health 2004/2005 business plan focused mainly on providing a single electronic record for health professionals across hospitals, primary care, and community services, it introduced a very limited degree of interactivity through the “chose and book” service [23]. At the same time, however, private companies were developing services that would enable patients to access their own electronic primary care record securely. In 2003, a private company started installing kiosks in GP surgeries that enabled patients to use fingerprint and pin authentication to gain access to their full GP electronic record [24]. By 2006, around 5000 patients had accessed their records in this way, and it was also possible to gain Web-based record access from home [24]. In 2007, the NHS introduced HealthSpace, a Web-based personal electronic health record, which enabled people to enter their health information and gain secure access to the summary care information in their GP record [25].

In 2010, the Department of Health outlined their vision of an information revolution incorporating Web-based access, giving people more control over their health care and improving choice [26]. The same year, the Royal College of General Practitioners published guidelines on enabling patients to access their electronic health records [27] and later published a more detailed “Road Map” on this topic [28]. Despite the British Medical Association’s concerns [29], the idea of Web-based patient access was now firmly on the UK government’s agenda, and in 2014, the National Information Board published a framework for action incorporating a vision stating,

In 2015, all citizens will have online access to their GP records and will be able to view copies of that data through apps and digital platforms of their choice...it is essential that citizens have access to all their data in health and care, and the ability to ‘write’ into it so that their own preferences and data from other relevant sources, like wearable devices, can be included... This framework prioritises comprehensive access—with the ability for individuals to add to their own records—by 2018.

30, p 21

Providing patients with the ability to write in their own health record will facilitate the collection of Patient-Reported Outcome Measures as advocated by Gensheimer et al [31].

## The Impact of Web-Based Access to Records

In 2012, to ascertain the impact of enabling patients to access their primary care record online, the Department of Health commissioned a systematic review of the evidence, supported by the Royal College of General Practitioners [32,33]; the review identified 17 randomized controlled trials, cohort, or cluster studies and summarized both the benefits and challenges of providing patients Web-based access to their record.

### Potential Benefits of Web-Based Access

Providing patients with Web-based access to their record has been shown to benefit both patients and clinicians. Web-based access enables patients to book appointments online, request repeat prescriptions, and view test results, letters, problems lists, and free-text GP entries [34], although there are wide variations in the degree of access provided by GP surgeries [35]. Patients who use Web-based access report higher levels of satisfaction [36] and improved communication with health care professionals [32]. Benefits to patients include being able to use the Web-based record as an aide memoir and help them prepare for their next appointment [35,37]. Patients like the convenience of Web-based access, stating that it saves time and money, and reduces the number of telephone calls and appointments required [32,35]. In addition, Web-based access can be empowering and increase patients’ feelings of autonomy, with one study noting that 77%-87% of patients with Web-based access feel more in control of their care [38]. Other benefits include enabling patients to share their records with family members or other health care providers, or to appoint a proxy to access their record [33]. Web-based access benefits both patients and clinicians in other ways such as improving self-care, increasing the uptake of preventive services, and enabling patients to spot medication errors and have them corrected [32]. The use of Web-based Patient-Reported Outcome Measures built into the patient record increases patients’ confidence in managing their condition and has been shown to reduce remission rates for conditions such as inflammatory bowel disease [39]. One study found that 70% of clinicians reported Web-based access improved trust, strengthened relationships, and enhanced decision making [38], while another found it reduced the annual number of visits and telephone calls [40].

### Challenges and Potential Negative Consequences

Despite the many benefits of enabling patients to access their record online, there are also a number of associated challenges. Clinicians have been especially resistant to opening up patient records for Web-based access owing to concerns that it will lead to an increased workload, cause unnecessary anxiety among patients, increase the likelihood of litigation, or challenge the current primary care business model [33]. Other concerns relate to security and confidentiality, equality issues (eg, literacy and internet access), risk of coercion, and information technology system compatibility [28]. The evidence regarding the impact of Web-based access on the clinicians’ workload is currently mixed, but there is inevitably an increase in the workload in the early transitional stage, including activities such as staff training [32]. As the patient record was not initially designed to be viewed by patients, the manner in which clinicians write in the notes will have to change if they are to be easily understood by a lay audience. One study, for example, noted that up to 36% of clinicians changed the record content to allow for Web-based access, and up to 21% reported spending more time writing notes [38]. Despite clinicians’ concerns regarding Web-based access causing anxiety among patients, leading to an increased risk of litigation, or data security breaches, a review of the studies, to date, has found little evidence these concerns are realized [33]. There is some evidence, however, that Web-based access could potentially lead to increases in health inequalities as those using Web-based access are more likely to be white, female, and middle class [32]. Although one might expect Web-based access to increase patient activation and, thus, improve health outcomes, less activated patients may be less likely to take advantage of Web-based access [41], thus potentially exacerbating health inequalities. Disappointingly, reviews of the literature, to date, reveal a lack of evidence for the impact of Web-based access on health outcomes [32,33], although an up-to-date systematic review is under way [42].

## Future Directions

We are still some way from realizing the National Information Board’s vision of all UK citizens having read and write access to their full primary care record through a variety of digital platforms that enable them to upload data from wearable devices. Enabling such read and write access could help GPs improve their understanding of the effect of disease and treatment on the everyday lives of patients [39]. The majority of GP practices offering Web-based access do so in a limited way, and although there are some notable exceptions [43], most do not allow access to the clinicians’ free-text entries [44]. As De Lusignan et al noted, there is a need for further research to determine “how the medical record might be redesigned to guide and teach patients in a way that promotes self-management and ultimately improves health” [33] (p 7). Such research should be multidisciplinary, drawing upon expertise from fields beyond medicine such as health psychology and human-computer interaction. We need to engage with health economists to ascertain the full economic potential of Web-based access and the impact it may have on the primary care business model. Although some studies using self-report measures exist [35,37,45], further research is also needed to examine how patients actually interact with their Web-based record and the functionality they would like to see. The impact of Web-based access on the patient-clinician relationship and the power dynamic is also worthy of further investigation, especially with regards to the impact of enabling access to the full free-text record. All of these issues underlie what must be our prime concern, and something for which the evidence is still limited, that is, how we can harness the potential of Web-based access to improve health outcomes. Patients’ expectations regarding access to their health information are changing, and the newly introduced General Data Protection Regulations [46] will undoubtedly shift the conversation further toward full unrestricted Web-based access. Clinicians will need to change how they view the patient record and learn to work with systems providers and patients to help instigate changes that will lead to improved health outcomes and increased savings for the NHS.

## Abbreviations

GP: general practitioner
NHS: National Health Service
